# Risk Factors, Health Effects and Behaviour in Older People during Extreme Heat: A Survey in South Australia

**DOI:** 10.3390/ijerph10126721

**Published:** 2013-12-03

**Authors:** Monika Nitschke, Alana Hansen, Peng Bi, Dino Pisaniello, Jonathan Newbury, Alison Kitson, Graeme Tucker, Jodie Avery, Eleonora Dal Grande

**Affiliations:** 1 Department for Health and Ageing, 11 Hindmarsh Square, Adelaide SA 5000, Australia; E-Mail: graeme.tucker@health.sa.gov.au; 2 School of Population Health, University of Adelaide, North Terrace, Adelaide SA 5005, Australia; E-Mails: alana.hansen@adelaide.edu.au (A.H.); peng.bi@adelaide.edu.au (P.B.); dino.pisaniello@adelaide.edu.au (D.P.); jonathan.newbury@adelaide.edu.au (J.N.); 3 School of Nursing, University of Adelaide, North Terrace, Adelaide SA 5005, Australia; E-Mail: alison.kitson@adelaide.edu.au; 4 Population Research and Outcome Studies, Discipline of Medicine, University of Adelaide, North Terrace, Adelaide SA 5005, Australia; E-Mails: jodie.avery@health.sa.gov.au (J.A.); eleonora.dalgrande@adelaide.edu.au (E.D.G.)

**Keywords:** heat wave, survey, behaviour, risk factors, heat-related illnesses, health knowledge, older people

## Abstract

Older people had a high incidence of hospitalisation during the 2009 heat wave in South Australia. We sought to explore resilience, behaviours, health risk factors and health outcomes during recent heat waves for a representative sample of independently living residents. A telephone survey of 499 people aged 65 years and over was conducted, and included both metropolitan and rural residences. A variety of adaptive strategies were reported, with 75% maintaining regular appointments and activities during the heat. However, 74% took medication for chronic disease and 25% assessed their health status to be fair to poor. In a multivariate model, factors associated with heat health outcomes included medication for mental health, heart failure, diabetes or respiratory health, reporting a reduced health status, use of mobility aids and being female. Compared with younger participants, those over 75 had more check-up calls and visits by family, friends and neighbours. However, confidence to call on support was associated with indicators of social isolation. The study indicates that older people are generally resilient, but interventions addressing multi-morbidity and medication interactions and social isolation should be developed.

## 1. Introduction

South Australia (SA, population: 1,662,200) has a Mediterranean climate with an average maximum temperature during January and February of 29 ºC and a minimum of 17 ºC. In summer 2009, SA experienced a prolonged heat wave of 13 days (26 January to 7 February 2009), reaching a record maximum temperature of 45.7 ºC, and six successive days over 40 ºC in the metropolitan area. The health impacts recorded for ambulance, hospital, emergency departments and death records for Adelaide, the capital city of SA, where 73% of the population reside, were considerable and by far exceeded the risks experienced during previous heat waves [1]. The burden of morbidity was particularly high in the older age groups, for whom direct heat-related hospital admissions were extremely high—up to 19 times higher compared to non-heat wave days during summer in the 75 years and over age group. There were also 140.5 excess heat-related emergency department presentations during the heat wave in the 65 years and over age cohort and a total excess of 304.4 across all age groups. Mental and renal health-related admissions were also predominantly increased in the older population. This disease pattern is in agreement with worldwide evidence from heat wave impacts and can be explained by physiological and cognitive contributing factors associated with ageing [2]. 

Health outcomes during heat waves in Australia are relatively well documented [3,4,5,6], but, there is scant Australia-specific information about the potential determinants of these heat health impacts. A number of surveys have been undertaken in other countries evaluating risks and risk perceptions of extreme heat and climate change in populations [7,8,9]. The objectives of such studies were to assess adaptive community capabilities which can provide essential information for preventive heat health plans [10]. Qualitative research of heat-susceptibility of older persons in SA, based on contributions from senior officers with medical, policy and emergency backgrounds, identified relevant health determinant domains that individually and interactively influence behavior and health outcomes of older people during heat waves [11]. Using this background information, a population based survey was undertaken in SA that investigated the adaptive capabilities in the elderly during extreme heat events. The aim of the survey was to gain relevant information that would enable future policies to address the potential risks and harness already existing adaptive strategies of older people to reduce further the preventable health problems caused by extreme heat.

## 2. Method

### 2.1. Data Collection

Ethical approval for the survey was obtained from both the SA Health and the University of Adelaide Human Research Ethics Committees. Survey information was gathered using computer assisted telephone (CATI) interviewing. 

All households in South Australia with a connected telephone and a telephone number listed in the current version of the electronic white pages were eligible for selection in the sample. Telephone numbers were drawn randomly. Only people aged 65 years and over were eligible for inclusion, and a sample of 4,200 households was initially drawn in order to reach the target number of 500. Screening calls were undertaken to obtain people in this age category. An initial survey question established type of residence. If responders lived in aged care facilities or nursing homes, the interview was terminated. The interview was also terminated if the respondent did not understand English or was not capable of completing the survey. If there were two or more people in the household aged 65 years and over, the selected respondent was the person with the most recent birthday. Selected people in households were not replaceable. Once a household was selected, there were up to 10 call-backs to busy or unanswered numbers at different times during the day, regardless of whether the target number of interviews had been reached. The questionnaire was piloted twice to ensure that all difficulties with questions were resolved. 

### 2.2. Survey Questions

The survey questions were mainly informed by our prior qualitative study, the results of which highlighted health factor domains including demographics, environment and housing, social connectedness, self-assessed health status, vulnerability and health problems during recent heat waves, heat health knowledge and resilience during heat waves [11]. 

### 2.3. Statistical Analysis

Data were weighted by the inverse of the individual’s probability of selection and then re-weighted according to “Estimated Residential Population” figures from the Australian Bureau of Statistics to ensure that the sample was representative of the target population in South Australia [12].

For statistical analysis, the results were grouped into variables informing about heat health behavior of older people, their potential risk factors (self-rated health status, chronic medication use and other health problems, social contacts and environmental factors), and outcome factors (health problems during recent heat waves, resilience factors, and social networks). The survey data were analysed using the survey estimators provided in Stata 12 [13]. In the first instance a descriptive analysis of the general and topic specific characteristics of participants was conducted. Differences in proportions were assessed for significance using chi-square tests and logistic regression.

Bivariate analysis was conducted using simple logistic regression analysis to explore the risk factors and their association with heat health outcomes. In order to ascertain independently associated factors, plausible influential risk variables with a *p*-value less than 0.2 at the bivariate level were simultaneously inserted into the multiple logistic regression models [14]. Using backward elimination, insignificant variables with the highest *p*-value were sequentially removed to yield the final set of risk factors associated with health outcomes and behaviours (*p* < 0.05). Confounding between predictors was assessed at each step. The Stata command “svylogitof” was used to assess goodness of fit of the design-based logistic regression model.

## 3. Results

### 3.1. Participation and Demographic Findings

Of the initial 4,200 randomly drawn households, a sample loss of 2,612 occurred. The reasons for the sample loss were non-residential numbers (36), disconnected numbers (838), fax/modem response only (30), and ineligibility due to age under 65 years or not residing in South Australia (1,708). 

The remaining sample of 1,588 constituted the eligible sample. There were 534 phone numbers not contactable after 10 attempts. Non-response was due to refusal of the interview (414), inability to speak English (73), illness/hearing impairment (55), termination of interview due to living in a facility (3), respondent unavailable (9), and not stating age (1). When subtracting the non-responders from the eligible sample, a participation rate of 47.7% was achieved and 499 interviews were conducted.

The mean age of the 499 eligible participants was 75.0 years (95% CI 74.4–75.8), with comparable numbers in the <75 years and ≥75 years age (Table 1) and gender categories. There were 341 metropolitan participants and 158 from rural areas. Table 1 shows demographic results for age, income, type of dwellings and living arrangements.

**Table 1:** Demographic outcomes and potential heat health risk factors (environment, socio-economic and health status). Weighted counts, percent and 95% CI.

Factors	Counts, N = 499*	Percent	95% CI
<75 Years of age	258.5	51.8	47.1; 56.5
≥75 Years of age	240.5	48.2	43.5; 52.9
Females	268.8	53.9	49.1; 58.6
Income			
Up to $20,000	163.0	32.7	28.4; 37.1
$20,000 to $60,000	158.5	31.8	27.4; 36.4
>$60,000	35.9	7.2	5.0; 10.2
Missing	141.6	28.4	24.4; 32.7
Dwellings			
House	410.7	82.3	78.6; 85.5
Unit	52.0	10.4	8.0; 13.4
Retirement village	34.2	6.8	4.9; 9.5
Other	2.1	0.4	0.1; 1.7
Living			
Alone	162.8	32.6	28.7; 36.8
With someone	336.2	67.1	62.9; 71.0

Note: * Discrepancies between sums of components and totals are due to rounding.

### 3.2. Heat Health Behaviour

Table 2 reports on the behaviour of older people during extreme heat. Generally older people changed their behavior by taking additional cool showers (26.8%), wearing summer clothing (91.4%), reducing sun exposure by closing blinds and curtains (91.6%), opening-up the house in the evenings (92.9%) to allow breezes through the house when it had cooled down and by drinking more fluid (91.4%). Only 21.5% of older people used the wet cloth method to cool down. While a large percentage of older people stated that they stayed indoors during heat (94.9%) and reduced outdoor activities (95.4%), they still maintained regular appointments (95.2%) and events (74%). Only 13.7% went to cooler places. Regular welfare phone calls or visits to respondents during heat waves were 30.1% and 17.6% respectively; people 75 years and over had more than twice the odds of receiving phone calls and being visited compared to those below 75 years of age (Odd Ratio (OR) 2.4; 95% Confidence Interval (CI) 1.5; 3.1; *p* < 0.001). 

**Table 2:** Behaviors of older people during extreme heat in SA. Weighted counts, percent and 95% CI.

Factors	Counts, N = 499	Percent	95% CI
Extra cool showers mostly to sometimes	133.6	26.8	22.9; 31.0
Wearing cooler clothes	456.3	91.4	88.3; 93.8
Reduction of physical activities	410.0	82.2	78.1; 85.6
Close blinds and curtains	457.2	91.6	88.7; 93.9
Use wet cloth on neck and face	107.3	21.5	18.0; 25.0
Stay indoors	473.8	94.9	92.5; 96.6
Reduction of outdoor activities	472.4	95.4	92.2; 96.4
Keep regular appointments	475.0	95.2	93.0; 97.7
Regular activities (shopping, sports, visits)	369.3	74.0	69.7; 77.9
Go to cooler places	68.3	13.7	10.8; 17.2
Open up the house in the evening	463.6	92.9	90.3; 94.8
Drinking more fluid	456.2	91.4	88.5; 93.7
Regular welfare phone calls during heat waves	150.2	30.1	26.0; 34.5
Regular welfare visits during heat waves	87.7	17.6	14.3; 21.4

### 3.3. Potential Heat Health Risk Factors

Table 3 indicates health-related pre-existing conditions, social and environmental risk factors. The majority of participants (75%) assessed their health to be good to excellent and 25% selected fair to poor as their status. Table 3 also shows the frequency distribution of specific medical conditions for which participants had taken medication on a regular basis. The conditions were selected on the basis of evidence of their possible association with increased risk during heat. More than half of the participants were taking medication for high blood pressure (56%), followed by medication for “other heart problems” (20.3%). Other listed health problems for which prescribed medication was taken were diabetes (16%), “kidney problems” (3%), respiratory disease (10%) and mental health-related conditions (10%). No medication was taken by 26% of the participants. More participants aged 75 years and older took medication for chronic conditions (78%) compared to those below the age of 75, although no significant difference was detected (71%) (OR 1.5; 95% CI 1.0; 2.4; *p* = 0.06). Mobility aids were used by 17% of older people and help in the household was needed by 38.4%. The percentage of people having contact with friends or relatives less than two times in the previous week by telephone, and in person, was 10.8% and 11.2% respectively, whilst 10% of people went out less than two times in the previous week.

**Table 3:** Potential heat health risk factors (environment, socio-economic and health status). Weighted counts, percent and 95% CI.

Factors	Counts, N=499	Percent	95% CI
Problems with access to transport/not self-driving			
Sometimes to most often	16.2	3.2	2.0; 5.1
Availability of blinds/awnings	305.1	61.1	56.4; 65.6
Use in summer	287.2	94.2	90.4; 96.5
Dwelling is insulated	446.2	89.4	86.4; 91.8
Dwelling has a/c	474.8	95.2	92.8; 96.8
Concerns about a/c costs	191.8	41.8	37.0; 46.7
Health status			
Good to excellent	373.4	74.8	70.6; 78.7
Fair to poor	124.7	25.0	21.1; 29.3
Don’t know	0.9	0.2	0.0; 1.3
Regular medication use			
Diabetes	79.0	15.8	12.7; 19.6
High blood pressure	278.2	55.7	51.0; 60.4
Heart failure	42.2	8.5	6.1; 11.6
Other heart problems	101.4	20.3	16.8; 24.4
Kidney	15.8	3.2	1.8; 5.5
Respiratory	48.8	9.8	7.4; 12.8
Mental health	51.9	10.2	7.7; 13.4
No medication	127.9	25.6	21.8; 30.0
Don’t know	13.9	2.8	1.6; 4.9
Use of mobility aid	82.8	16.6	13.5; 20.2
Help needed with household tasks?	191.5	38.4	33.9; 43.1
Social Contacts (friends, relatives)			
Phone contact < 2 times per week	52.7	10.8	7.9; 13.9
Talked in person < 2 times per week	55.7	11.2	8.4; 14.7
Visited other people < 2 times per week	50.1	10.1	7.6; 13.3

Only 3.2% of the not self-driving older people claimed to have problems with access to transport. A very high percentage of people (95.2%) had air conditioning (a/c) in their house, but 41.8% mentioned concerns about the running cost. When blinds or awnings were available (61.1%), a majority of the participants (94.2%) used them in summer.

### 3.4. Heat Health Outcome Factors

Table 4 displays the results for heat health outcome factors. Specific symptoms experienced during recent extreme heat events are listed. The highest prevalence was for loss of balance (12.9%), followed by shortness of breath (12.3%), heat stress (12.0%) and headache (11.4%). Seventeen percent of respondents had experienced one or more of these symptoms during the extreme 2009 heat wave. More than half of the respondents (51.4%) had major concerns about the heat and 13.7 % felt more at risk than other people of their own age. A small percentage of respondents were not confident calling on friends or neighbours (14.6%) or family (7.6%) for help during extreme heat. Over half the respondents stated that they did not change their behavior after receiving heat health messages and 20.9% did not recall extreme heat warnings. Heat health warning recall was significantly lower for the 75 years and over age group (70%) compared to those below 75 years of age (87%) (OR 2.8; 95% CI 1.7; 4.5; *p* < 0.001).

**Table 4:** Outcome factors (heat health, resilience, social connectedness and recall). Weighted counts, percent and 95% CI.

Factors	Counts, N = 499	Percent	95% CI
Heat health outcomes			
Anxiety	26.7	5.3	3.7; 7.7
Loss of balance-dizzy	64.3	12.9	10.1; 16.3
A fall	15.3	3.1	1.7; 5.4
Headache	56.9	11.4	1.7; 5.4
Shortness of breath	61.2	12.3	9.5; 15.6
Heat stress	59.7	12.0	9.3; 15.3
Heart condition	22.5	4.5	2.9; 7.0
Something else	25.1	5.0	3.4; 7.4
Any of these symptoms in 2009	84.4	16.9	13.7; 20.7
Resilience during extreme heat			
Some to major concerns about heat health	233.7	51.4	46.7; 56.1
No to little concern	256.7	46.8	42.2; 51.6
More at risk than person of the same age	68.3	13.7	10.7; 17.3
Less to same risk	409.8	82.1	78.2; 85.5
Not confident during extreme heat			
To cal l on friends or neighbours	73.7	14.8	11.7; 18.5
Call on family	38.0	7.6	5.4; 10.6
Behavior after heat health warnings			
No behavior change	266.9	53.5	48.7; 58.2
No recall of heat health messages	104.4	20.9	17.3; 25.0

### 3.5. Multivariate Analysis

Table 5 shows the risk factors (from Table 3) that were related to outcome factors (from Table 4) in the multivariate analysis. The results indicate that relevant pre-existing illnesses requiring prescribed medications (for mental health, heart failure, diabetes and respiratory health) were associated with health outcomes reportedly experienced by the survey participants in recent heat waves. Overall, health status was an important factor indicating that people whose health status was fair to poor had an increased risk of experiencing symptoms during extreme heat than those who felt healthier. 

**Table 5:** Multivariate relationships between heat health outcomes and risk indicators. Odds Ratios, 95% CI and *p*-values.

Outcomes	Risk Indicator Variables	OR (95% CI) *p*-value
Anxiety	Medication (M) mental health	6.1 (2.5, 15.1) **
	Use of mobility aid	3.2 (1.3, 7.8) *
Loss of balance/dizzy	Reduced health status	2.6 (1.3, 5.3) *
	Income	1.8 (1.1, 3.0) *
	M for heart failure	2.6 (1.0, 7.1) ^#^
A fall	M for diabetes	4.3 (1.3, 14.4) *
Headache	Reduced health status	2.0 (1.0, 3.8) *
	M for mental health	2.7 (1.2, 6.0) *
Shortness of breath	Reduced health status	2.4 (1.3, 4.6) *
	M for heart failure	3.5 (1.7, 7.3) *
	M for respiratory problems	3.5 ( 1.6, 7.4) *
	Help in household needed	1.9 (1.0, 5.7) ^#^
	Using mobile aids	2.9 ( 1.5, 5.8) *
Heat stress	M for mental health	4.6 (2.2, 9.4) **
	Use of mobile aids	2.3 (1.2, 4.4)
Heart condition	M for heart failure	34.1 (11.5, 101.6) **
	Help in household needed	2.7 (0.9, 8.0) ^#^
Any of the above symptoms (yes compared to no)	Gender (female)	1.6 (1.02, 2.4) *
	Reduced health status	2.2 (1.4, 3.5) *
	M for heart failure	2.4 (1.2, 4.8) *
	Use of mobile aids	1.8 (1.0, 3.0) *
Symptoms during the 2009 heat wave (yes compared to no)	M for mental health	2.9 (1.4, 6.0) *
	Using mobile aids	3.0 (1.7, 5.3) **
Extreme heat a concern (yes compared to no)	M for respiratory problems	1.9 (1.0, 3.6) ^#^
	M for mental health problems	2.7 (1.4, 5.4) *
More at risk during extreme heat (yes compared to no)	Reduced health status	2.3 (1.3, 4.4) *
	Use of mobile aids	2.2 (1.1, 4.3) *
Recall of health messages (No compared to yes)	Age	1.1 (1.1, 1.1) **
	M for Diabetes	2.2 (1.2, 4.1) *
	Contact in person less than twice in the last week	1.7 (1.2, 5.9) *
Not changed behavior after heat warnings (No compared to yes)	Decreasing income	1.6 (1.1, 2.2) *
	M for mental health issues	3.1 (1.4, 6.7) *
Not confident calling on Friends/neighbours (No compared to yes)	Contact in person less than twice in the last week	2.8 (1.4, 5.7) *
	Gender (females)	1.8 (1.01, 3.3) *
Not confident to contact family member (No compared to yes)	Phone contact with people less than twice in the last week	6.1 (2.4, 15.9) **
	Decreased income	2.9 (1.2, 7.0) *

Notes: **^#^** *p* < 0.1; * *p* < 0.05; ** *p* < 0.001.

Females were more at risk of having had one or more symptoms during recent heat waves. Survey responders who were at risk of ill health during the 2009 heat wave were also more likely to use mobility aids and take mental health related medication.

The confluence of reduced health status, use of mobility aids and taking medication for respiratory and mental health problems was also relevant in relation to responders for whom extreme heat is of great concern and who believed themselves to be more at risk during extreme heat than the average person of their age. 

Increasing age, taking medication for diabetes and having reduced social contact were associated with a reduced recall of health messages. Those with reduced income and taking mental health-related medication were more likely to not change their behaviours after extreme heat warnings. Those that had reduced social contacts and females were less confident to ask for help from friends and neighbours than those having more frequent contacts. People with reduced social contact and reduced income were less confident to contact family members.

## 4. Discussion

This is the first study to focus on heat-related risk factors for an older, but independently living population in South Australia. This subpopulation was chosen because older people had a high incidence of hospitalisation during the 2009 heat wave in South Australia and are known to be more at risk when exposed to extreme heat [1]. Considering that climate change-related increases in temperatures may also increase the frequency and the duration of extreme heat days, it is important to investigate factors that may increase the risks of adverse health outcomes in Australia and in turn inform prevention measures. There have been a number of studies which have explored risk factors following major heat waves in other countries [15,16,17,18], but the identified country specific factors may not necessarily coincide with those relevant for Australia. For example, Vandentorren reported of risks to the French population during the 2003 heat wave associated with upper floor living and low ventilation rates [19]. In SA, very few multi storey houses exist and a/c prevalence is high. This study aimed to glean information from older independent living residents about their behaviours, perceptions and pre-existing illnesses using a representative population sample. 

The chronological age cut-off point of 65 years for defining older people has been chosen based on the current cultural interpretation of older age which is largely consistent with the official retirement age. The categorisation into <75 and ≥75 years of age is not based on any medically-based cut-off point, but provides some interesting insights into changes in behaviours, resilience and health outcomes in chronological terms. A further consideration was the much higher risk for the 75 years and over age group for direct heat-related emergency and hospital admission compared to the 65–74 year age group during the 2009 heat wave in Adelaide [1].

Gender, area (metropolitan *vs.* country) and age of the SA general population were used for calculation of survey weights. When the weighted and unweighted percentages of age group and area were compared, only minor discrepancies were seen indicating that the survey results reflect the age group distribution in the general population. In relation to gender, the survey results show that more females participated in the study or were more likely to be at home during survey calls than males. To further assess the generalizability to the general older population in SA, the frequency distribution of the health status variable in this survey was compared with a large 2011 SA survey in the 65 and over age group indicating very similar frequency distributions for the health status variable [20].

The survey indicated that there is a high prevalence of older people who undertake adaptive behaviours during heat waves. The majority of responders made good decisions that have the potential to modify heat exposure. Nevertheless, this still leaves a sizeable percentage of older people who may not consider themselves at risk and hence have opted to answer questions about preventive behaviours in the negative. The majority of older people seem to keep their regular appointments and undertake their regular activities during a heat wave which has the potential to adversely affect their health in extreme circumstances. Eighty two percent did not feel that they were more at risk compared to their age peer group and 47% felt that extreme heat is of no or little concern for their health; while only 26% of the participants did not take chronic disease-related medication. In a survey of older people (aged 72–94) in London (UK), participants did not consider themselves to be old and vulnerable, even if they suffered from chronic diseases which have the potential to interact with heat exposure [7]. This positive, but potentially uninformed thinking may also play a role in this survey, where a sizable percentage of older people with chronic disease did not undertake appropriate heat health behaviour and did not change their behaviour after extreme heat warnings. These findings may warrant special attention when formulating health promotion strategies.

The survey found that during recent heat waves, 30% of older people were contacted regarding their welfare by phone and 18% were visited. Considering the recent intense heat waves in SA, this percentage could be increased by encouraging families, friends and the wider community to routinely include welfare calls during extremely hot and long heat waves. Also, fifteen percent of older people did not feel confident to call on friends or neighbours, and 8% to call on family members if they needed help in the event of a major heat wave. This lack of confidence was significantly associated in the multivariate analysis with having minimal phone and personal contacts in the week prior to the survey indicating some level of social isolation among older people in South Australia.This is an area of concern, particularly as social isolation puts people at risk during extreme heat, especially if they have pre-existing ill health and may not be aware of health warnings. Overseas studies have clearly shown that social connectedness is an indicator for reduced risk of mortality during extreme heat [17,21]. On the positive side, people 75 years and over received more check-up calls and were visited more often during heat waves than those below 75 years of age indicating heat health awareness by family, friends and neighbours. 

Presence of an a/c has been shown to be highly protective of heat-related illness and mortality in studies overseas [16,17]. While the survey results indicate that 95% of South Australians older people own an a/c, 42% of the respondents had fair to major concerns about the cost of using an a/c. A/C can provide effective shelter from heat, but can be expensive to operate. Notwithstanding, older people should be encouraged to use cooling during extreme heat events and save energy during other times. Installation of blinds, awnings or outdoor shutters has been recommended for keeping houses cool without ongoing energy costs [22]. In this study, 61% used these less costly cooling methods.

While 76% of older people remembered some heat health messages, this recall was significantly reduced in the 75 and older age group challenging the ‘one size fits all’ approach of health warnings. The majority of the participants heard warnings and recommendations on the radio and television (data not shown); both were voted to be the best means of communicating warnings, but short message service (SMS) was also considered (15%). Interestingly, only 9% of participants considered newspapers to be the best means of effective communication. 

Fair to poor health status was identified in 25% of the study population. Seventy four percent of those were taking medication for one or more chronic diseases, with high blood pressure and heart-related issues being the most prevalent diseases, followed by diabetes, mental and respiratory health issues. These chronic conditions and associated medication have the potential to interact adversely with extreme heat [23]. Increasing age is also by itself associated with physiological changes that decrease the ability to feel the heat and to sweat, but perspiration is required to lower body temperature when ambient heat levels exceed 34 °C [24]. In this survey, 9% did not increase their intake of fluid during hot weather and 31% of those stated that this was due to not feeling thirsty (data not shown).

Health effects most commonly reported during recent heat waves by participants were loss of balance, followed by shortness of breath, heat stress and headache. In the multivariate analysis chronic disease-related use of medication, use of mobile aids, need of help in the household and reduced social contacts were all significantly related to these health effects experienced during recent heat waves. These relationships are in line with findings from a number of overseas studies. Presence of chronic diseases, reduced social contacts and reduced mobility were all significantly associated with morbidity and mortality during severe heat waves in France, USA and Canada [15,16,19,21].

The study has some shortcomings. The cross-sectional design only allowed for a snapshot of perceptions, behaviours and health effects, and a cohort approach may provide a greater understanding of the causal relationship between indicators and heat health outcomes over a summer period. The summer prior to the survey was unusually mild and may have influenced respondents’ recall of behaviours during hot weather. The study may also be biased towards the less vulnerable; as those unable to participate due to health problems are not included in this survey. The non-English speaking community was also excluded, as well as those not connected to a landline. 

## 5. Conclusions

The target population used adaptive behaviours, but there was also a sizable group which did not see themselves as potentially vulnerable and did not engage in behaviour changes. Considering that 25% of this representative population of people 65 and over in SA indicate a reduced health status and 74% take medication for chronic health conditions, the results may indicate that the participants may underestimate potential consequences of ill health during extreme heat. This is supported by their self-reported health problems, especially indicated by taking cardiovascular-related medications which were strongly correlated with health problems experienced during recent heat waves. Other risk factors impacting on having experienced health issues were the need for mobility aids and needing help in the household. The results of this survey indicate a number of risk factors amenable to prevention through targeted education and policy development.
